# Assessment of Knowledge and Attitudes Over Time in Postacute COVID-19 Environments: Protocol for an Epidemiological Study

**DOI:** 10.2196/52114

**Published:** 2023-11-23

**Authors:** Iván Martínez-Baz, Vanessa Bullón-Vela, Núria Soldevila, Núria Torner, David Palma, Manuel García Cenoz, Glòria Pérez, Cristina Burgui, Jesús Castilla, Pere Godoy, Angela Domínguez, Diana Toledo

**Affiliations:** 1 Instituto de Salud Pública de Navarra Pamplona Spain; 2 Centro de Investigación Biomédica en Red de Epidemiología y Salud Pública Madrid Spain; 3 Instituto de Investigación Sanitaria de Navarra Pamplona Spain; 4 Universitat de Barcelona Barcelona Spain; 5 Agència de Salut Pública de Barcelona Barcelona Spain; 6 Universitat Pombeu Fabra Barcelona Spain; 7 Universitat de Lleida Catalonia Spain; 8 Institut de Recerca Biomèdica de Lleida Catalonia Spain

**Keywords:** COVID-19, knowledge, attitudes, household contact, vaccination, preventive measures, survey

## Abstract

**Background:**

Globally, COVID-19 is in transition from the acute pandemic phase into a postacute phase, and special attention should be paid at this time to COVID-19 control strategies. Understanding public knowledge and attitudes plays a pivotal role in controlling COVID-19’s spread and provides information about the public’s adherence to preventive and control measures.

**Objective:**

This study protocol describes the planning and management of a survey to investigate the persistent or changing trends in knowledge and attitudes regarding COVID-19, vaccination, and nonpharmaceutical preventive measures among COVID-19 cases’ household contacts aged 18 years and older, after the acute phase of the pandemic in Catalonia and Navarre in Spain. The secondary objectives include investigating the rate of secondary transmission in households, taking into account the demographic characteristics, clinical manifestations, and preventive measures toward COVID-19.

**Methods:**

A telephone questionnaire was designed to assess the changing trends in knowledge, preventive measures, and attitudes toward COVID-19 in 3 rounds (after identification as a household contact, 3 months later, and 6 months later). The questionnaire was developed following an extensive literature review and through discussions with a panel of experts who designed and assessed the validity of the questionnaire in terms of relevance, consistency, completeness, and clarity. The questionnaire consists of the following 7 sections: social and demographic characteristics (ie, gender, age, educational level, and workplace), comorbidities and risk factors (according to the recommendations from the COVID-19 vaccination strategy), epidemiological data (ie, exposure time, relationship with index cases, and frequency of use of nonpharmaceutical preventive measures), COVID-19 vaccination status (ie, the number and date of doses received), knowledge and attitudes toward COVID-19 (assessed using a 5-point Likert scale—totally agree, agree, neither agree nor disagree, disagree, and totally disagree), and sources of information (including traditional mass media, social media, and official sources).

**Results:**

A pilot study was performed in May 2022 to evaluate the questionnaire with 22 household contacts. Preliminary findings indicated that the questionnaire was feasible and acceptable in the general population. The average response time was 15 minutes, with greater variations in responses by older participants. After the pilot study, recruitment of participants began and is expected to be completed at the end of the year 2023, after which the final results will be available in 2024.

**Conclusions:**

Despite the low transmission levels of SARS-CoV-2 and the relaxation of containment measures, the implementation of the survey during the postacute phase will provide valuable insight to assist public health decision-making and control the transmission of SARS-CoV-2 and other respiratory viruses, thereby attenuating the negative effects of COVID-19 at individual and population level.

**International Registered Report Identifier (IRRID):**

DERR1-10.2196/52114

## Introduction

### Overview

The COVID-19 pandemic has demonstrated that infectious diseases are a continuous challenge for public health. Prevention and control measures to diminish the burden of SARS-CoV-2 infection in the population have changed over time, from exclusively implementing nonpharmaceutical interventions to extensive vaccination programs [1-3].

According to the last report of the European Centre for Disease Prevention and Control (ECDC) [4], many European countries are currently de-escalating their response measures due to the transitional process of the acute pandemic phase into the postacute phase. However, the ECDC encourages governments and national authorities to monitor pandemic trends and key indicators, focused on preventing and managing not only SARS-CoV-2 variants but also the long-term health consequences due to COVID-19 [4]. Although many studies on knowledge and attitudes related to COVID-19 have been published [5,6], there are still several knowledge and behavioral gaps, which can serve as a guide to address future emergencies relating to SARS-CoV-2 variants and other pandemics, considering that knowledge, attitude, and behavior can change over time [6].

It is generally recognized that populations’ levels of knowledge and attitudes regarding COVID-19 and its preventive measures are important factors for controlling transmission during the pandemic [4]. In the acute phase of the COVID-19 pandemic, some studies evaluating knowledge and attitudes have been conducted worldwide, both in the general population and in specific groups [7-10]. However, many gaps in the knowledge and practices concerning COVID-19 need to be filled. For example, in a multinational sample, including 22 countries, authors showed that almost one-third of participants did not know that infected individuals can be asymptomatic, increasing their risk of exposure to COVID-19 [5]. In addition, a higher correlation was found between attitudes and practices compared to knowledge and practices, indicating that knowledge itself is not enough and that effective interventions to improve practices should aim to encourage both adequate knowledge and positive attitudes [5]. Other studies demonstrate that the probability of transmission is higher for people who meet the criteria of close contacts, due to their exposure to COVID-19 cases with laboratory-confirmed infection. Close contacts include household contacts, among whom exposure is usually more intense and repeated; therefore, the risk of transmission may be high, even in the absence of other factors that enhance transmission [11,12].

At this time, the population has access to accurate real-time information on the evolution of the pandemic and the recommendations to follow, which could be reliably disseminated by health authorities and official institutions through various secure communication channels [1-3]. Nevertheless, the population is also exposed to other media sources that spread unreliable and contradictory information about the evolution of the pandemic, causing confusion regarding the measures to be followed. In addition, it must be noted that the absence of knowledge and inappropriate beliefs about the importance and best methods of prevention may increase the risk of infection [13]. In this context, data about gaps in knowledge and behavioral information is crucial to the prevention and management of future resurgences of SARS-CoV-2 variants.

### Objectives

The main objective of this study is to investigate the persistent or changing trends in knowledge and attitudes about COVID-19, vaccination, and nonpharmaceutical preventive measures in household contacts of COVID-19 cases after the acute phase of the pandemic in Spain.

The secondary objectives of the study are as follows:

- To compare the characteristics of index cases and primary cases of COVID-19
- To investigate the rate of secondary transmission, taking into account factors such as age, sex, clinical manifestations, and preventive measures related to the first case in the household
- To determine the rate of secondary transmission in households with primary cases of COVID-19 caused by new variants of SARS-CoV-2 and the existence of vaccination failures

## Methods

### Study Design

This study is a prospective epidemiological study using a telephone survey addressed to household contacts of confirmed COVID-19 cases in 2 regions of Spain: Navarre and Catalonia. The telephone survey is being conducted in 3 rounds: the first round is directly after the identification of an eligible contact; the second round will be 3 months later, and the third round will be 6 months after the initial contact. These interviews are currently in progress, with most participants having completed the second interview as of the date of this paper’s submission.

### Ethical Considerations

The study was conducted in accordance with the Declaration of Helsinki and approved by the Institutional Ethics Committee of the University of Barcelona (protocol code IRB00003099) on March 2, 2023. Oral consent is requested from participants by the Institutional Ethics Committee of the University of Barcelona, and an informative document about the study is sent by email to whoever requests it.

### Participants

The recruitment of household contacts is currently being conducted in 9 primary health care centers (1 from Navarre and 8 from Catalonia) associated with COVID-19 cases. The inclusion criteria are as follows: patients who are positive for COVID-19 (cases) and household contacts aged ≥18 years at the time of selection, who agree to participate in the study and provide oral consent. Those aged <18 years and those with the presence of severe and uncorrectable cognitive, visual, or hearing impairments that hinder the participants’ ability to complete interviews are excluded from the study.

### Organization and Coordination Activities

The study was managed by Centro de Investigación Biomédica en Red de Epidemiología y Salud Pública through the University of Barcelona and the Institute of Public Health of Navarre, in coordination with the epidemiological surveillance services of both regions. The main function of the project coordination committee is to supervise and monitor the adequate application of the methodology and working plan to achieve the project objectives. The selection of COVID-19 cases is being carried out by primary health care professionals who also report the COVID-19 cases to the epidemiological surveillance services. COVID-19 cases are being selected if they have had at least 1 eligible household contact who could be identified. All household contacts of COVID-19 cases reported to the epidemiological surveillance services in both regions are selected to participate in this study. All participants are informed of the objectives of the study and invited to participate. Individuals included in the study have provided prior oral consent to be contacted and are informed that all information will be treated anonymously in the subsequent statistical analyses.

**Figure 1:**
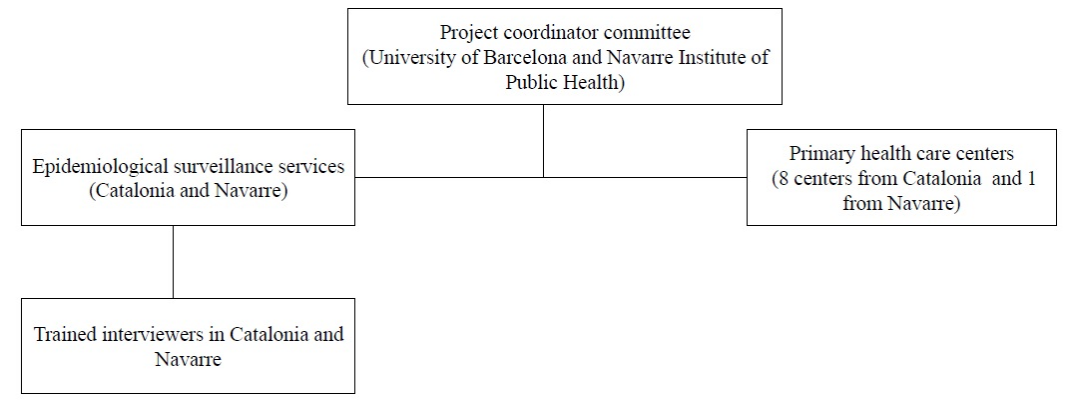
The organizational structure of the project team.

Qualified interviewers are conducting the surveys by telephone in both regions (Figure 1). Complete information on the coordination of activities is accessible to all researchers involved in the study through a manual of procedures developed by the coordinating committee.

During the initial stages of screening, household contacts of COVID-19 cases will be identified through the epidemiological surveillance services or primary health care centers from Catalonia and Navarre. Trained study personnel will perform a formal screening evaluation to confirm eligibility for the study, which will take place after the patient has provided oral informed consent.

### Data Collection

#### Literature Review and Design of the Questionnaire

The first step of the questionnaire design, an exhaustive literature review, was carried out by the coordination committee [14]. After this literature review, the questionnaire was structured, taking into account the recommendations against COVID-19 provided by the World Health Organization, ECDC, and the Spanish Ministry of Health [1-3,15]. The research team, composed of professionals with experience in epidemiological and public health research, held a series of preliminary meetings to develop all sections of the survey, including the selection of questions and the number of items included. Discussion of the survey focused on the relevance, consistency, completeness, and clarity between linked questions, the length of the survey, and the overlap of questions between sections of the survey. All the sections of the questionnaire, especially the items related to knowledge and attitudes, were adapted from instruments used in other publications related to the study topic, specifically the questionnaires used by Xu et al [16] and Geldsetzer [17]. The questions were adapted to the specific circumstances of the Spanish population, ensuring conceptual and semantic equivalence. Following an iterative process that resulted in several major revisions of the first draft.

The survey was developed in Spanish, and it consisted of 7 sections: social and demographic information, comorbidities and risk factors, epidemiological information, COVID-19 vaccination status, knowledge concerning COVID-19 and its preventive measures, attitudes toward COVID-19 and its preventive measures, and participants’ sources of information about COVID-19.

The questionnaire also includes demographic information, variables related to comorbidities, and COVID-19 vaccination status, which could be validated through electronic health record linkage with the regional vaccination registers and databases of epidemiological surveillance.

#### Questionnaire Structure

##### Social and Demographic Characteristics

Demographic variables include participants’ gender; age; educational level (according to the International Standard Classification of Education by the United Nations Educational, Scientific and Cultural Organization); workplace (remote work, on-site work, or both); and the severity of the index case, including whether or not their treatment involved hospitalization.

##### Comorbidities and Risk Factors

Information about the prior presence of major chronic conditions is being collected according to the recommendations from the COVID-19 vaccination strategy, including chronic obstructive pulmonary disease, coronary heart disease, stroke, diabetes mellitus, immunodeficiency (eg, HIV/AIDS and immunosuppression), chronic kidney disease, chronic liver disease, neurological and psychiatric disease, and cancer [18]. Information about risk factors, including tobacco use (smoker, former smoker, or nonsmoker), obesity (BMI>30, BMI>40), and hypertension, is also obtained from the household contacts. These data are collected in dichotomous variables according to the presence or absence of each one of them.

##### Epidemiological Information

The epidemiological section registers data related to whether the participants have had close contact with the index case (first positive case in the household); the exposure time; the relationship with the index case (ie, partner, father, or mother); whether they have shared a vehicle or bedroom with the index case; and whether the contact has symptoms, and if so, which ones. Other data collected include the frequency of use of nonpharmaceutical preventive measures at home (eg, ventilation, use of mask, distance, and frequency of hand washing); the results of, and the type of COVID-19 test (eg, polymerase chain reaction test or rapid antigen test) they may have had after the positive test results of the index case; and any previous COVID-19 infection, including details about severity, possible hospitalization, and any other relevant details.

##### COVID-19 Vaccination Status

This section provides data concerning COVID-19 vaccination history, including the date of administration and brand of each dose. The data provided by the participants in this section can be validated by the vaccination records. To evaluate vaccination hesitancy, if the participant has not received any vaccine, they are asked for possible reasons for their lack of willingness to receive it.

##### Knowledge Concerning COVID-19 Disease and Its Preventive Measures

In this section, the patients’ knowledge of COVID-19 is evaluated, considering that adequate knowledge about COVID-19 infection, severity, and preventive measures are important factors associated with the decision of vaccine uptake, and therefore, the spread of infection [19]. This section consists of 6 questions, and participants are asked about COVID-19 modes of transmission and preventive measures during a situation of COVID-19 circulation (Table 1). The knowledge about COVID-19 disease and preventive actions, such as vaccination and nonpharmaceutical measures, are assessed using a 5-point Likert scale (totally agree, agree, neither agree nor disagree, disagree, and totally disagree) [10].

**Table 1:** Items concerning knowledge of COVID-19 disease and its preventive measures.

	Totally disagree	Disagree	Neither agree nor disagree	Agree	Totally agree
All people who become ill with COVID-19 develop severe cases.	—^a^	—	—	—	—
Asymptomatic persons diagnosed with COVID-19 can transmit the infection.	—	—	—		—
Persons diagnosed with COVID-19 can transmit infection despite vaccination.	—	—	—	—	—
The COVID-19 virus is spread by respiratory droplets from infected individuals when coughing, sneezing, talking, laughing, and singing.	—	—	—	—	—
Handwashing is important to reduce the risk of contracting COVID-19.	—	—	—	—	—
To prevent transmission of COVID-19, measures such as wearing face masks and avoiding crowds in enclosed spaces should be maintained.	—	—	—	—	—

^a^To be filled.

##### Attitudes Toward COVID-19 Disease and Its Preventive Measures

This section consists of 6 questions, evaluating attitudes toward COVID-19 vaccination; stigma against symptomatic individuals; and the effects of COVID-19 on daily life, considering the current situation (Table 2). For this section, participants are being asked to state their level of agreement with specific attitudes and preventive actions related to COVID-19 again, expressed on a 5-point Likert scale [7].

**Table 2:** Items related to attitudes toward COVID-19 disease and its preventive measures.

	Totally disagree	Disagree	Neither agree nor disagree	Agree	Totally agree
I consider myself susceptible to developing a severe disease if I become ill with COVID-19.	—^a^	—	—	—	—
I consider that my inner circle has complied with the preventive measures to avoid becoming ill with COVID-19.	—	—	—	—	—
I consider that my inner circle has complied with the vaccination recommendations given by the health authorities.	—	—	—	—	—
I consider that it is better to develop immunity by getting sick with COVID-19 than by getting vaccinated.	—	—	—	—	—
It is convenient for the general population to wear a facemask correctly (covering the nose and mouth) to prevent COVID-19 in crowded, closed environments.	—	—	—	—	—
I consider that COVID-19 had a negative influence on my daily life.	—	—	—	—	—

^a^To be filled.

##### COVID-19 Information Sources

Additionally, the survey includes a section regarding the most common source of information on COVID-19 disease and the preventive measures consulted by the participants. The survey contains a list of possible information sources as the primary source used by the participant and has the following options: nonofficial sources, such as traditional mass media (eg, television and written or web-based press); social media (eg, Instagram, Facebook, and Twitter); and official sources, such as medical professionals and government or other official sources (eg, World Health Organization, ECDC, or Spanish Ministry of Health).

## Results

### Pilot Test

The content validity of the questionnaire was evaluated in a pilot study performed in Navarre, consisting of a sample of 22 household contacts during the period between April and June 2022. In the pilot test, participants were presented with a set of items and questions by a trained staff interviewer. The interviews were performed by telephone, and all observations were collected during this phase. No changes to the initial questionnaire’s items related to the knowledge and attitudes toward COVID-19 disease, its preventive measures, or information sources were needed. The pilot test confirmed that the questionnaire had established content and was well understood and concise.

The final questionnaire consisted of 23 questions distributed in 7 sections (described in the previous section). The average response time was 15 minutes, with older participants exhibiting a more noticeable variation in their responses.

### Evaluation of the Trends in Knowledge and Attitudes Over Time

To evaluate the persistence or changing trends in knowledge and attitudes about COVID-19, vaccination, and nonpharmaceutical preventive measures, the telephone survey of household contacts is being conducted in 3 rounds, at different epidemiological moments. The first round is done just after their identification as a household contact, and the second and third rounds are done 3 and 6 months later, respectively.

**Figure 2:**
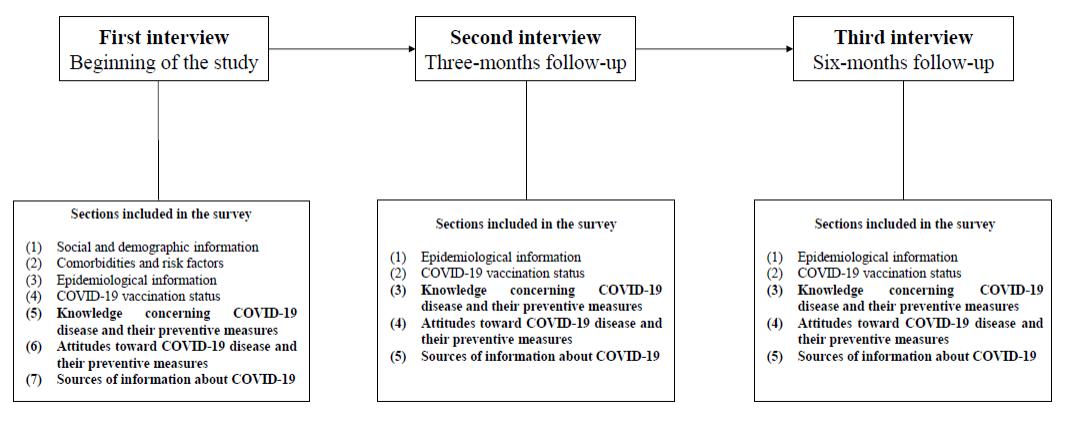
Study design and follow-up timetable of the study.

In the first round, all the information from the questionnaire is collected, thus obtaining reference information at the time of contact with the index case. In the 3-month and 6-month follow-up rounds, we will inquire whether they have had any change in their COVID-19 vaccination status or whether they have become infected by the SARS-CoV-2 since the last call; we will also inquire about various items related to knowledge and attitudes about COVID-19 and its preventive measures as well as the information sources upon which they rely (Figure 2). The background information is not collected again, as it is not subject to change over time. After the pilot study, participant recruitment began and is expected to be completed by the end of 2023. The final results will be available in 2024.

## Discussion

### Principal Findings

This study will provide valuable insights into the knowledge and attitudes of household contacts of confirmed COVID-19 cases, after the acute phase of the pandemic, toward the disease and its preventive measures, including vaccination and nonpharmaceutical preventive measures, and will allow for evaluating the trends in these data over time.

At this moment, this study is still in the recruitment process. It must be mentioned that this study is being carried out within the framework of the postacute phase of COVID-19, where infections continued to decrease. Therefore, including a preliminary analysis of this questionnaire, considering the recruited population at this moment, could provide biased results. However, despite the abundance of information available about infection and preventive measures for COVID-19, studies have shown that there are still some gaps related to the use and maintenance of preventive measures during this transitional phase of the pandemic, especially in households, where the probability of SARS-CoV-2 transmission is higher [11,12]. Preventive measures, such as vaccination and nonpharmaceutical measures, are of special relevance for controlling this infection. More than 50% of transmission chains are generated in households, and the use of nonpharmaceutical preventive measures before the occurrence of cases is probably limited [20].

Some studies reported that nonpharmaceutical public health measures, such as physical distancing and wearing masks, had a strong impact on person-to-person COVID-19 transmission at the individual level [21,22]. Funke et al [22] evaluated the containment of nonpharmaceutical measures during the first 3 pandemic waves in countries from North and South America, Europe, and Asia-Pacific. Results showed a dual cause-effect channel where nonpharmaceutical interventions had a direct impact on several health outcomes and an indirect effect on voluntary changes in social distancing. Some authors have identified that accurate knowledge is essential in managing epidemic situations caused by infectious agents and that nonadherence to nonpharmaceutical preventive measures is likely to be the consequence of a knowledge gap [23-25].

Detecting the weak points in the population’s attitudes and knowledge regarding the studied preventive measures, as well as monitoring the persistent or changing nature of adherence to them over time, is important for planning strategies to improve prevention and health promotion. It also serves as a benchmark for future epidemics of respiratory viruses that affect the population every winter. This issue is particularly relevant in the current scenario in which vaccines do not totally reduce the risk of transmission [26].

The current situation of the COVID-19 pandemic, following the acute phase during the emergence of the Omicron wave, indicates a notable decrease in the number of new cases, which many might interpret as a sign of the end of the pandemic. Corresponding with this, we have seen a change in the surveillance and control strategy against COVID-19 implemented by the Spanish government and health authorities. This shift is accompanied by reductions in the compulsory and recommended use of nonpharmaceutical interventions, such as the compulsory use of face masks [27]. This will naturally result in the lower use of these measures in the population and possibly a shift in their beliefs regarding these measures and COVID-19 itself.

A key factor is to understand how containment measure strategies impact pandemic stages to minimize the risk of further waves of SARS-CoV-2. Cascini et al [28] compared the impact of COVID-19 containment measures in 5 European countries on the epidemic curves. Authors indicated 3 main factors that affected COVID-19 transmission. First, the time when preventive measures were adopted—as long as these measures were implemented early, they showed better results; second, the duration of containment measures; and finally, the number of COVID-19 cases before relaxing containment measures. It is important to emphasize that the effectiveness of preventive measures depends on their adherence.

The attitude of acceptance of the recommended measures is essential to control the transmission of SARS-CoV-2 and reduce its incidence, especially in situations in which these measures are extended over time with different intensities, and as a consequence, the population is liable to reduce their compliance [29,30]. Knowledge and attitudes are important cognitive keys in public health regarding prevention and health promotion. Other factors that influence noncompliance may include a range of beliefs about the causes of the disease, knowledge of the identification of symptoms, available methods of treatment, consequences, and possible long-term complications [25]. Some studies related to knowledge and attitude regarding COVID-19 have been conducted, but most of them were performed on the general population [31-34], in which the risk of transmission is lower than in a closed environment, such as a household. Evidence from these studies on knowledge and attitudes during epidemics allows us to recognize and identify key attitudes and practices to prevent the spread of the disease. Consequently, several policies and political interventions have been established and reinforced to optimize the management of the COVID-19 pandemic by health authorities.

We believe that it is important to monitor such changes over time and assess any knowledge gaps to understand both the current situation and how such knowledge and beliefs evolve over time. The low current incidence of COVID-19 cases reflects a complex matrix of influences rather than being a reliable sign of the end of the pandemic. A combination of high vaccination coverage, an increase in immunity in the population generated by natural infections, and the cumulative effects of compulsory and recommended preventative measures [35-37], along with other factors, create the situation we currently experience. In the case that a new outbreak may occur, information about the ongoing beliefs, preventative practices, and knowledge gaps in the population will be invaluable in designing new strategies to combat the disease.

A strength of this study is that the coordination committee is composed of professionals with expertise in communicable diseases and vaccination research. They have been involved in the surveillance and monitoring of the infection in Catalonia and Navarre since the beginning of the COVID-19 pandemic, which facilitates the implementation of the study in both regions and allows adequate management of fieldwork, with a unified work protocol. Another strength of the study is that it is being carried out in 2 regions of Spain (Catalonia and Navarre) that present different situations of SARS-CoV-2 household transmission, increasing the representation of the Spanish population. Additionally, conducting a longitudinal study assessing changing beliefs and knowledge in the community during the postacute phase of the pandemic provides, in our view, an essential dimension to understanding the social landscape in which the measures are being applied. The combination of these 3 factors makes the results of this study uniquely valuable in disease prevention.

This study has some limitations. First, it is being carried out at a time when the epidemiological situation presents a low incidence of COVID-19 cases, which could alter the sample size of the contacts to follow. Therefore, we have planned to contact every single reported case during the study period and carry out an exhaustive census of household contacts to maximize participation. Second, it is a survey-based study, and the information will be self-reported, so information biases could possibly be introduced in the outcome variables and information about vaccination status. However, demographic characteristics, comorbidities, and COVID-19 vaccination status can be verified with electronic health records and vaccination registers. Third, the relaxation of the containment measures might have an impact on the attitudes toward COVID-19 over time, but our study could provide relevant information on attitudes toward preventive measures during this postacute phase of the pandemic [4].

### Conclusions

The development of a questionnaire to assess knowledge and attitudes about COVID-19, vaccination, and nonpharmaceutical preventive measures over time after the acute phase of the pandemic will provide valuable insights regarding the control of infection in the population, in a low transmission scenario. This questionnaire will provide information about knowledge and attitudes over time and will be important for public health decision-making during the relaxation of containment measures in the event of an increase in COVID-19 cases or a new health emergency due to respiratory viruses.

## Appendix

Multimedia Appendix 1 Peer-review report from the Centro de Investigación Biomédica en Red Epidemiología y Salud Pública (CIBERESP) (Madrid, Spain).

## Abbreviations

ECDC: European Centre for Disease Prevention and Control

## Data Availability

Study data will be available upon reasonable request from the corresponding author. The data will not be published on a public platform for ethical reasons. Data will be anonymized if an access request is received.
