# Supplementary material for: Assessment of Knowledge and Attitudes Over Time in Postacute COVID-19 Environments: Protocol for an Epidemiological Study
Source: JMIR Res Protoc. 2023 Nov 23;12:e52114. doi: 10.2196/52114 (PMC10704316; doi:10.2196/52114)
Supplement: Multimedia Appendix 1 [file resprot_v12i1e52114_app1.pdf]

INTRAMURAL CALL FOR CIBERESP PROJECTS 2022

Project evaluation: “Knowledge and attitudes of household contacts of COVID-19 cases towards the disease and its prevention through vaccination and non-pharmacological measures”. PIs: Diana Toledo Zabaleta and Iván Martínez Baz.

Please, write the PI's name or ID number project (you can find it in the folder name or in the proposal document)	A.1.- Evaluation of the RESEARCH TEAM (0-10)	A.2.- Evaluation of 5 PAPERS of PI in the LAST 5 YEARS (0-10)	A.3.- Evaluation of 5 PAPERS of coPI in the LAST 5 YEARS (0-10)	Brief Reasoning of the Quantitative Evaluation Proposed	Puntuación A (30%)	B.1.- SCIENTIFIC QUALITY OF THE PROPOSAL (0-10)	B.2.- QUALITY OF THE DESIGN AND METHODS (0-10)	B.3.- FEASIBILITY AND SPECIFICATION OF THE WORK PLAN (0-10)	B.4.- APPLICABILITY OF THE TRANSFER PLAN (0-10)	B.5.- POTENTIAL RESULTS AND THEIR ALIGNMENT WITH CIBERESP (0-10)	B.6.- REQUESTED BUDGET (0-10)	Brief Reasoning of the Quantitative Evaluation Proposed	Puntuación B (65%) Según cuestionario = (B.1 * 1.5) + (B.2. * 2) + B.3 + B.4 + B.5	C COLLABORATION WITH OTHER AREAS CIBER (0-5) 0=No; 3=project including at least 1 group from other areas CIBER; 5=project including groups from at least 2 other areas CIBER	SUBTOTAL Puntuación bloque A + Puntuación bloque B, que según cuestionario usado = [(B.1 * 1.5) + (B.2. * 2) + (B.3. * 0.5) + (B.4 * 0.5) + B.5 + B.6] + Puntuación por colaboración de otras áreas C	IF THE TOTAL SCORE IS AT LEAST OF 70 points [Si total suma evaluacion de cada par/2 >= 70 (mayor o igual que 70 ptos)] SUMA PARES/2	YOUTH (PI/coPI) Add 5 points if co-PI<45 years; add 7 points if PI<45 years; add 10 points if both PI and co-PI <45 years	TOTAL POINTS (110 ptos max.)
P9	9	8	9	The research team involves investigators from four CIBERESP groups, but does not appear to involve other CIBERs. Some of these investigators are involved in a project funded by the ISCIII aimed at the investigation of COVID-19 secondary transmission and vaccine effectiveness. The 5 publications listed by both PI's show experience in working on surveillance, risk factors, and treatment and prevention of COVID-19, influenza and other infectious diseases. In both cases, the publications are in international journals with relatively high impact. The lead PI is lead author in only one of the five papers listed, while the co-PI is lead author in four of the five. None of their publications appear to be primarily focused on the evaluation of attitudes and perceptions (the main goals of the present proposal), although other members of the research groups have published on this topic (e.g., attitudes and perceptions among health professionals and pharmacists).	26	9	7	8	5	10	10	The proposal is an ancillary study of a funded ISCIII project that adds a component not addressed in the parent study. The study aims have public health significance for the management of the ongoing SARA-CoV-2 pandemic and (possibly) future pandemics. The methods are appropriate for the study aims, including a longitudinal component that will allow for the evaluation of changing attitudes and perceptions over time. It is not entirely clear how multiple infections in a given households will be handled in the analysis. Low response rates and the self-report nature of the information collected are potential limitation, as acknowledged by the investigators. Another potential limitation is the generalizability of the findings with respect to future epidemics (or future stages of the COVID-19 epidemic) as the social/political context evolves over time. The budget seems appropriate for the proposed aims and procedures.	60,5	0	86,5			
P9	9	7	9	The team is excellent. The PIs have been productive and show great expertise and productivity in COVID-19	25	7	7	6	5	8	7	I have some concerns regarding doing a survey via the phone for this project. It could result in high non-response, which could be differential by opinion regarding vaccines. It is also not clear how this survey would contribute to a future larger study. The study motivation spent too much time with general aspects of the COVID pandemic that were not necessary. The cost of the phone calls is not budgeted.	50,5	0	75,5			
																81	10	91

RESOLUCIÓN CONVOCATORIA INTRAMURAL PROYECTOS CIBERESP 2022

Con fecha 17 de septiembre de 2021 se publicó la Convocatoria Intramural de Proyectos de Investigación 2022 de 2 años de duración para grupos pertenecientes a CIBERESP, con el objetivo de fomentar la colaboración entre grupos y áreas, el establecimiento y la consolidación de sinergias entre investigadores de distintas instituciones pertenecientes a CIBERESP, así como facilitar el liderazgo de investigadores jóvenes CIBERESP.

Dicha convocatoria se cerró el 17 de octubre con la presentación de 13 propuestas por parte de los grupos CIBERESP.

Según el presupuesto disponible para la convocatoria 2022 el número de proyectos que pueden ser financiados como máximo es de 6.

La evaluación de los proyectos ha sido realizada por pares por evaluadores externos miembros del Comité Científico Asesor Externo (CCAE) de CIBERESP. En la evaluación realizada se han puntuado los siguientes aspectos:

- Interés y calidad de la propuesta.
- Perspectiva de salud pública.
- Alineación con las líneas estratégicas de CIBERESP.
- Idoneidad del/de la IP y co-IP (publicaciones que justifican su liderazgo en el proyecto propuesto).
- Grado de colaboración entre grupos CIBERESP, así como con otras áreas CIBER (ISCIII).
- Calidad y factibilidad del plan de transferencia.

Además, aquellos proyectos cuyo IP y/o co-IP es joven (<45 años) han recibido una valoración adicional. Siendo la puntuación máxima que puede obtener el proyecto 110 puntos.

Tras analizar los resultados de la evaluación, la Dirección Científica de acuerdo con el Comité de dirección de CIBERESP resuelven financiar los siguientes proyectos:

Proyecto	Título	Puntos	Financiación total
P5	Population-based genomic epidemiology study for tailored strategies for surveillance and control of tuberculosis (TB-SEQ)	103,25	50.000,00 €
P12	The impact of COVID-19 pandemic and their restrictions on the chronification of benzodiazepine use	99,00	45.500,00 €
P6	EPI (Embarazo y Primera Infancia) DATA	94,00	50.000,00 €

P4	IMMAGE – Opportunistic body composition assessment from routine imaging data in patients with multiple myeloma	91,75	50.000,00 €
P10	Measuring the real impact of West Nile virus infection in Spain	91,00	48.000,00 €
P9	Knowledge and attitudes of household contacts of COVID-19 cases towards the disease and its prevention through vaccination and non-pharmacological measures	91,00	49.100,00 €

Los proyectos tendrán una duración de 2 años. El **periodo de ejecución** del primer año comienza tras la resolución de la convocatoria hasta 31 de diciembre de 2022. El periodo de ejecución del segundo año abarca desde 1 de enero a 31 de diciembre de 2023.

El **resultado final** del proyecto podrá ser científico y/o tecnológico (un programa, una aplicación etc.); en este último caso el producto final estará disponible en la web de CIBERESP.

A cada proyecto concedido se le asignará un número identificador que deberá figurar entre las fuentes de financiación de todos los productos derivados del mismo.

Al final de la primera anualidad se presentará una **memoria de progreso** de resultados del proyecto. La continuidad de la financiación en la segunda anualidad está supeditada a la evaluación favorable de la primera memoria.

Al final de los dos años se presentará la **memoria final**. El/la IP y co-IP se comprometen a presentar los resultados en la Jornada CIBERESP.

En Madrid, a 23 de febrero de 2022.

MARINA ANUNCIACION| POLLAN| SANTAMARIA

Firmado digitalmente por MARINA ANUNCIACION| POLLAN|SANTAMARIA
Fecha: 2022.02.23 12:17:22 +01'00'

ALONSO CABALLERO JORDI - 37732070H

Firmado digitalmente por ALONSO CABALLERO JORDI - 37732070H
Nombre de reconocimiento (DN): c=ES, serialNumber=IDCES-37732070H, givenName=JORDI, sn=ALONSO CABALLERO, cn=ALONSO CABALLERO JORDI - 37732070H
Fecha: 2022.02.23 13:27:39 +01'00'

Dra. Marina Pollán Santamaría
Directora Científica de CIBERESP

Dr. Jordi Alonso Caballero
Subdirector Científico de CIBERESP

Comissió de Bioètica
Universitat de Barcelona

Dictamen favorable

La Comissió de Bioètica de la Universitat de Barcelona (CBUB), en sessió ordinària del dia 5 de novembre de 2021, va avaluar els aspectes metodològics, ètics i legals del projecte de recerca "Conocimientos y actitudes de los contactos domiciliarios de casos de Covid 19 frente al Sars-Cov 2 y su prevención durante la vacunación", emetent observacions enfront d'aquest.

El 24 de febrer de 2022, la investigadora principal, la doctora Diana Isabel Toledo Zavaleta, va enviar la documentació requerida en la reunió celebrada el mateix dia que ha estat avaluada per la Secretaria de la CBUB, obtenint dictamen favorable.

En Barcelona, a 2 de març de 2022.

JORDI GARCIA FERNANDEZ - DNI 35030054L (TCAT)

Firmado digitalmente por JORDI GARCIA FERNANDEZ - DNI 35030054L (TCAT)
Fecha: 2022.03.10 18:51:24 +01'00'

Dr. Jordi García Fernández
Vicerector de Recerca
President de la Comissió de Bioètica
Universitat de Barcelona

Institutional Review Board (IRB00003099)

Instituto de Salud Pública y Laboral de Navarra
Nafarroako Osasun Publikoaren eta Lan Osasunaren Institutua

Doña María Ángeles Nuin Villanueva, Directora Gerente del Instituto de Salud Pública y Laboral de Navarra, autoriza la realización de los proyectos titulados:

- **“Factores asociados a la transmisión de SARS-CoV-2 en los convivientes de Cataluña y Navarra y efectividad de las vacunas y de las medidas no farmacológicas para reducir la transmisión”** que ha obtenido financiación a través de la Acción Estratégica en Salud 2021.

- **“Knowledge and attitudes of household contacts of COVID-19 cases towards the disease and its prevention through vaccination and non-pharmacological measures”** que ha obtenido financiación a través de la Convocatoria Intramural de Proyectos CIBERESP 2022.

Ambos proyectos están relacionados entre sí, y están bajo la coordinación de la Sección de Vigilancia Epidemiológica y Vacunaciones de este instituto.

Pamplona, 11 de marzo de 2022

NUIN VILLANUEVA MARIA ANGELES - DNI 18200573Y

Firmado digitalmente por NUIN VILLANUEVA MARIA ANGELES - DNI 18200573Y
Nombre de reconocimiento (DN): c=ES, o=GOBIERNO DE NAVARRA, ou=CERTIFICADO ELECTRONICO DE EMPLEADO PUBLICO, ou=ISPLN, ou=N648750, serialNumber=IDCES-18200573Y, sn=NUIN VILLANUEVA, givenName=MARIA ANGELES, cn=NUIN VILLANUEVA MARIA ANGELES - DNI 18200573Y
Fecha: 2022.03.11 11:23:18 +01'00'

Fdo.: Doña María Ángeles Nuin Villanueva
